# Locoregional interventional therapy for hepatocellular carcinoma: radiologic and clinical factors predictive of untreatable progression and time to untreatable progression

**DOI:** 10.3389/fphar.2024.1413696

**Published:** 2024-07-23

**Authors:** Zijun He, Xueying Zhang, Yucong Zhang, Jian Kong

**Affiliations:** ^1^ The Second Clinical Medical College, Jinan University, Shenzhen, China; ^2^ Department of Radiation Oncology, Shenzhen People’s Hospital (Second Clinical Medical College of Jinan University, First Affiliated Hospital of Southern University of Science and Technology), Shenzhen, China; ^3^ Department of Interventional Radiology, Shenzhen People’s Hospital (Second Clinical Medical College of Jinan University, First Affiliated Hospital of Southern University of Science and Technology), Shenzhen, China

**Keywords:** hepatocellular carcinoma, untreatable progression, time to untreatable progression, transcatheter arterial chemoembolization, ablation, initial response, best response

## Abstract

**Objective:**

In this retrospective cohort study, independent risk factors that influence untreatable progression (UP) and time to UP (TTUP) in patients with hepatocellular carcinoma (HCC) after locoregional interventional therapy were examined. The effects of initial response and best response on UP occurrence and TTUP after locoregional interventional therapy were evaluated.

**Methods:**

Data were collected from HCC patients who were initially treated with the drug-eluting beads–transcatheter arterial chemoembolization (DEB-TACE) procedure at our hospital from January 2017 to December 2022. Modified response evaluation criteria in solid tumors (m-RECIST) was used to evaluate the radiologic response of tumors. Logistic regression analysis was used to analyze the risk factors for UP in patients, and Cox regression analysis was used to discover independent variables that influenced TTUP.

**Results:**

A total of 93 patients who initially underwent the DEB-TACE procedure were included. Subsequent to initial treatment, 50 patients continued with DEB-TACE treatment, while 43 received DEB-TACE and sequential thermal ablation treatment. The probability of developing UP was 82.8% (n = 77). Furthermore, 49 (52.7%) patients achieved an initial response, and 70 (75.3%) achieved the best response. Multivariate logistic regression analysis confirmed three independent risk factors of UP, namely, age (odds ratio [OR]: 0.950, *p* = 0.044); initial response (OR: 0.177, *p* = 0.020); and treatment regimen (OR: 7.133, *p* = 0.007). Multivariate Cox regression found that total bilirubin (hazard ratio [HR]: 1.029, *p* = 0.002), tumor distribution (HR: 1.752, *p* = 0.034), Subjective Angiographic Chemoembolization Endpoint (SACE) classification (HR: 0.668, *p* = 0.043), number of tumors (HR: 1.130, *p* = 0.004), initial response (HR: 0.539, *p* = 0.019), and treatment regimen (HR: 4.615, *p* < 0.001) were independent variables that influenced TTUP.

**Conclusions:**

Age, initial response, and treatment regimen significantly affected the occurrence of UP in HCC patients. Initial response, SACE classification, treatment regimen, total bilirubin, number of tumors, and tumor distribution were significantly correlated with TTUP. The initial response following locoregional interventional therapy had greater effects on UP occurrence and TTUP than the best response.

## 1 Introduction

Primary liver cancer ranks fourth among the most common malignancies worldwide and is the second leading cause of cancer-related deaths in China, of which hepatocellular carcinoma (HCC) accounts for 75%–85% of all primary liver cancers (Sung et al., 2021; Xia et al., 2022a). As the clinical symptoms of early liver cancer are unremarkable, most patients are diagnosed at intermediate to advanced stages and thus miss the optimal time window for a radical procedure. Locoregional interventional therapy for HCC includes transcatheter arterial chemoembolization (TACE), ablation, and radioembolization. Currently, many guidelines recommend TACE as the standard of care for patients with intermediate-stage HCC.

TACE treatment regimens can be divided into conventional TACE (C-TACE) and drug-eluting beads–TACE (DEB-TACE). Studies have shown that the objective response rate and the incidence of postoperative complications of DEB-TACE were superior to those of C-TACE (Poon et al., 2007; Golfieri et al., 2014). Most HCC patients require multiple TACE treatments to achieve tumor remission, which risks the worsening of hepatic function. Therefore, physicians usually make decisions for subsequent TACE treatment in clinical practice based on on-demand treatment, that is, based on tumor response following TACE, the Child–Pugh score, and patients’ clinical presentations after TACE (Terzi et al., 2012).

The Japan Society of Hepatology (JSH) was the first to propose the concept of TACE refractoriness to decide whether TACE should be discontinued and changed to systemic treatment (Kudo et al., 2021). Recently, some clinical studies have begun studying TACE refractoriness (Wang et al., 2021; Zou et al., 2021). Interestingly, the JSH included new intrahepatic tumors in the criteria for TACE refractoriness and recommended discontinuation of TACE treatment. However, subsequent TACE treatment can usually lead to tumor responses in the form of new lesions, which is considered a treatable progression (Kudo et al., 2020b). Instead, when the tumor shows severe progression and the continuation of TACE no longer benefits the patient or improves survival, the treatment regimen should be promptly changed. This progression is defined as an untreatable progression (UP) (Bruix et al., 2011; Forner et al., 2018). According to some guidelines and clinical studies, there are some differences in the UP definition, but most of them describe worsening hepatic function, increasing clinical stage, and altered radiology findings (Wang et al., 2020; Ren et al., 2022, 2018). The concept of UP is not only limited to TACE treatment but also suitable for all Barcelona Clinic Liver Cancer (BCLC) stages and all treatment regimens (Reig et al., 2022). UP represents the failure of the current treatment regimen, indicating that the treatment strategy should be promptly changed.

Overall survival (OS) is the gold standard for measuring patient prognosis. However, the successive use of multiple treatments increases the number of confounding factors, possibly affecting the evaluation of response to certain treatment regimens. Therefore, the use of OS for the evaluation of HCC response to locoregional interventional therapy remains challenging, and there is an urgent need for rational surrogate endpoints that can evaluate treatment outcomes.

Time to UP (TTUP) primarily describes the time from the start of a treatment to UP in patients. One study showed that TTUP was highly correlated with OS but had a shorter follow-up duration and fewer confounding factors. Thus, TTUP may be a surrogate endpoint for OS to evaluate the efficacy of locoregional interventional therapy (Labeur et al., 2019). Another study indicated that during locoregional interventional therapy, the tumor response during different treatment cycles could also significantly affect patient prognosis (Georgiades et al., 2012).

Therefore, this study focused on HCC patients who received DEB-TACE-based locoregional interventional therapy (DEB-TACE or DEB-TACE + sequential thermal ablation) to explore the risk factors of UP after locoregional interventional therapy and variables that influence TTUP, with the aim to provide recommendations for the prompt switching of treatment regimens. We also analyzed the effects of initial response and best response on UP and TTUP after locoregional interventional therapy.

## 2 Materials and methods

### 2.1 Study subjects

This study enrolled HCC patients initially treated with DEB-TACE at our hospital between January 2017 and December 2022. The inclusion criteria were as follows: 1) clinical or pathologic diagnosis of HCC according to the standard for diagnosis and treatment of primary liver cancer (2022 edition) published by CSCO (Commission, 2022); 2) age between 18 and 85 years; 3) BCLC stage B or stage A but unable or unwilling to undergo radical treatment; 4) Child–Pugh hepatic function grade A or B; and 5) the US Eastern Cooperative Oncology Group (ECOG) score of 0 points. The exclusion criteria were as follows: 1) history of surgery or radiotherapy following the initial HCC treatment or systemic treatment; 2) incomplete baseline and follow-up data; 3) invasive or diffuse HCC; 4) no arterial enhancement in the lesion or maximum lesion <1 cm; 5) other severe comorbidities or metabolic disorders, such as severe renal and cardiovascular diseases or severe diabetes mellitus; 6) spontaneous liver cancer rupture and bleeding; and 7) previous C-TACE treatment or ablation within 6 months prior to the DEB-TACE procedure. This study was approved by the ethics committee of our hospital [LL-KY-2024011-01], and the need for informed consent from patients was waived. The study is registered at the Chinese Clinical Trial Registry (ChiCTR 2200060448).

### 2.2 DEB-TACE procedure

A femoral artery puncture was carried out using the Seldinger technique under local anesthesia. The catheter sheath was inserted over a guidewire. Abdominal aortography was performed to observe anatomical changes in the hepatic artery, followed by selective hepatic arteriography to comprehensively evaluate the blood vessels nourishing the tumor and simultaneous supraselective cannulation of these blood vessels. Under fluoroscopy, 1–2 vials of 50 mg/vial of 70–150, 100–300, and 300–500 μm of pirarubicin-coated drug-eluting beads (CalliSpheres^®^ beads; Hengrui Medical, Suzhou, China) or DC^®^ beads (Biocompatibles UK Ltd., Farnham, United Kingdom) were slowly injected, with the chemoembolization dose rationally determined based on the tumor burden. During chemoembolization, all nourishing blood vessels were embolized to the greatest possible extent to devascularize the tumor. Subjective Angiographic Chemoembolization Endpoint (SACE) (Lewandowski et al., 2007) was used to evaluate the degree of tumor embolization. Following the procedure, patients were treated with routine hepatoprotection, gastroprotection, analgesia, antiemetics, and other symptomatic treatments. Next, dynamic contrast-enhanced computed tomography/magnetic resonance imaging (CT/MRI) was performed every 6–8 weeks to evaluate the tumor response. Patients entered the follow-up observation stage on complete response (CR) after the first cycle of the DEB-TACE procedure. If residual tumors, tumor enlargement, or new tumors were detected by dynamic contrast-enhanced CT/MRI, the subsequent treatment regimen was formulated after discussion by the multidisciplinary team. Patients were divided based on the treatment regimen. Patients who received DEB-TACE treatment alone were included in the monotherapy group, while those who underwent DEB-TACE and sequential ablation treatments were included in the combined group. Patients were followed up until UP, missed follow-up, or death. The follow-up period ended on September 30, 2023.

### 2.3 Study endpoints

The primary endpoint was UP. The BCLC guidelines (Reig et al., 2022), the European Association for the Study of the Liver (2018), and existing literature (Bruix et al., 2011; Forner et al., 2014; Wang et al., 2020; Ren et al., 2022) were used as references to define UP as follows: 1) existing tumor enlarged by >20% or new intrahepatic tumors (>1 cm) and subsequent locoregional interventional therapy (such as DEB-TACE or ablation) did not induce objective response in progressed tumors, absence of vascular invasion, or extrahepatic metastasis; 2) the presence of extrahepatic metastasis or vascular invasion; 3) the Child–Pugh hepatic function score ≥10 or ECOG score ≥1; and 4) the target tumor is still in stable disease (SD) after at least two continuous intervention treatments. The secondary endpoints were TTUP and initial response and best response of the tumor after treatment. TTUP was defined as the time from the start of treatment to UP (Hsu et al., 2012; Kudo et al., 2022). Table 1 shows the specific definitions of initial response and best response.

**TABLE 1:** Definitions of tumor characteristics.

Relevant parameters	Definition and classification
HCC dynamic contrast-enhanced CT type (Kawamura et al., 2010)	Type 1: homogeneous enhancement with no increase in arterial blood flow Type 2: homogeneous enhancement with increased arterial blood flow Type 3: heterogeneous enhancement with a diaphragm-like structure Type 4: heterogeneous enhancement pattern with irregular ring-like structures The enhancement types were divided into two categories, namely, types 1 + 2 and types 3 + 4 (Hu et al., 2020)
Hepatic function albumin–bilirubin (ALBI) score (Zheng et al., 2017)	1 g bilirubin (μmol/L) × 0.66+ albumin (g/L) × −0.085 scored as “1” for values of ≤ −2.60, “2” for > −2.60 and ≤ −1.39, and “3” for > −1.39
Tumor distribution (Vesselle et al., 2016)	Classified according to Couinaud’s hepatic segments. Tumors limited to the right lobe, left lobe, or middle lobe were defined as monolobar tumors; otherwise, tumors were classified as multilobar tumors
SACE classification (Lewandowski et al., 2007)	Level I: normal arterial blood flow and decreased tumor staining Level II: decreased arterial blood flow and tumor staining Level III: decreased arterial blood flow and tumor staining not observed Level IV: arterial blood flow and tumor staining not observed
Prothrombin (PT) time prolongation score (Durand and Valla, 2008)	1 point: PT prolonged by 1–3 2 points: PT prolonged by 4–6 3 points: PT prolonged by >6
Six-and-twelve tumor burden grouping (Wang et al., 2019)	Longest diameter of largest lesion + number of lesions = tumor burden; divided into three groups based on the sum ≤ 6; or > 6 but ≤ 12; or > 12
Initial response (Choi et al., 2014)	Presence of initial response: achieved CR or PR at follow-up after the first TACE procedure Absence of initial response: maintained SD or progressed to PD at follow-up after the first TACE procedure
Best response (Kim et al., 2015)	Presence of best response: best radiologic result was CR or PR during locoregional interventional therapy; if the same objective response was maintained during this period, the initial tumor response and time point were considered as best responses (Xia et al., 2022b) Absence of best response: the objective response of the tumor was maintained at SD or PD during repeated treatments
Tumor objective response (based on m-RECIST) (Lencioni and Llovet, 2010)	Complete response (CR) Partial response (PR) Stable disease (SD) Progressive disease (PD)

### 2.4 Data collection

Baseline clinical data and radiologic data of patients were collected to screen for UP risk factors post-procedure and variables that influenced TTUP. The collected data included the following 23 markers: sex (male/female), age, etiology (HBV/other), the BCLC stage (A/B), the Child–Pugh grade (A/B), the albumin–bilirubin (ALBI) grade (1/2 + 3), serum albumin, total bilirubin level, platelet count, alpha fetoprotein (AFP) (≤400/>400)), PT prolongation time score, drug-eluting bead particle size, tumor distribution (monolobar/multilobar), vascular lake (present/absent), capsule (present/absent), type of enhancement (1 + 2/3 + 4), SACE classification at the first TACE procedure, maximum tumor diameter, number of tumors, six-and-twelve tumor burden grouping (the sum is ≤ 6; or > 6 but ≤ 12; or > 12), treatment regimen (monotherapy/combined), initial response (present/absent), and best response (present/absent). Table 1 shows the clinical characteristics and radiologic lesion feature definitions of patients. All data were assessed by two radiologists blinded to the clinical data. The modified response evaluation criteria in solid tumors (m-RECIST) (Lencioni and Llovet, 2010) were used to evaluate the tumor radiologic response after treatment.

### 2.5 Statistical analysis

Categorical variables were expressed as frequency and percentage, and continuous variables were expressed as mean and standard deviation. Univariate logistic regression analysis was used to screen out risk factors of UP. In addition, the candidate variables with statistically significant differences were included in the multivariate logistic analysis to determine the independent predictors of UP. Kaplan–Meier survival curves were used to analyze median TTUP, and the log-rank test was used to compare inter-group differences. Univariate Cox regression was used to determine variables that influenced TTUP before multivariate Cox stepwise regression was used to confirm the independence of each variable and to calculate their hazard ratio (HR) and 95% confidence interval (CI). Cohen’s kappa was used to determine subjective radiologic agreement. Cohen’s kappa values were indicated as poor agreement, <0; slight agreement, 0.0–0.20; fair agreement, 0.21–0.40; moderate agreement, 0.41–0.60; substantial agreement, 0.61–0.80; and almost perfect agreement, 0.81–1.0. All the above data were processed using SPSS Statistics 26 (IBM Corporation, Armonk, NY, United States), and *p* < 0.05 indicated a statistically significant difference.

## 3 Result

**FIGURE 1:**
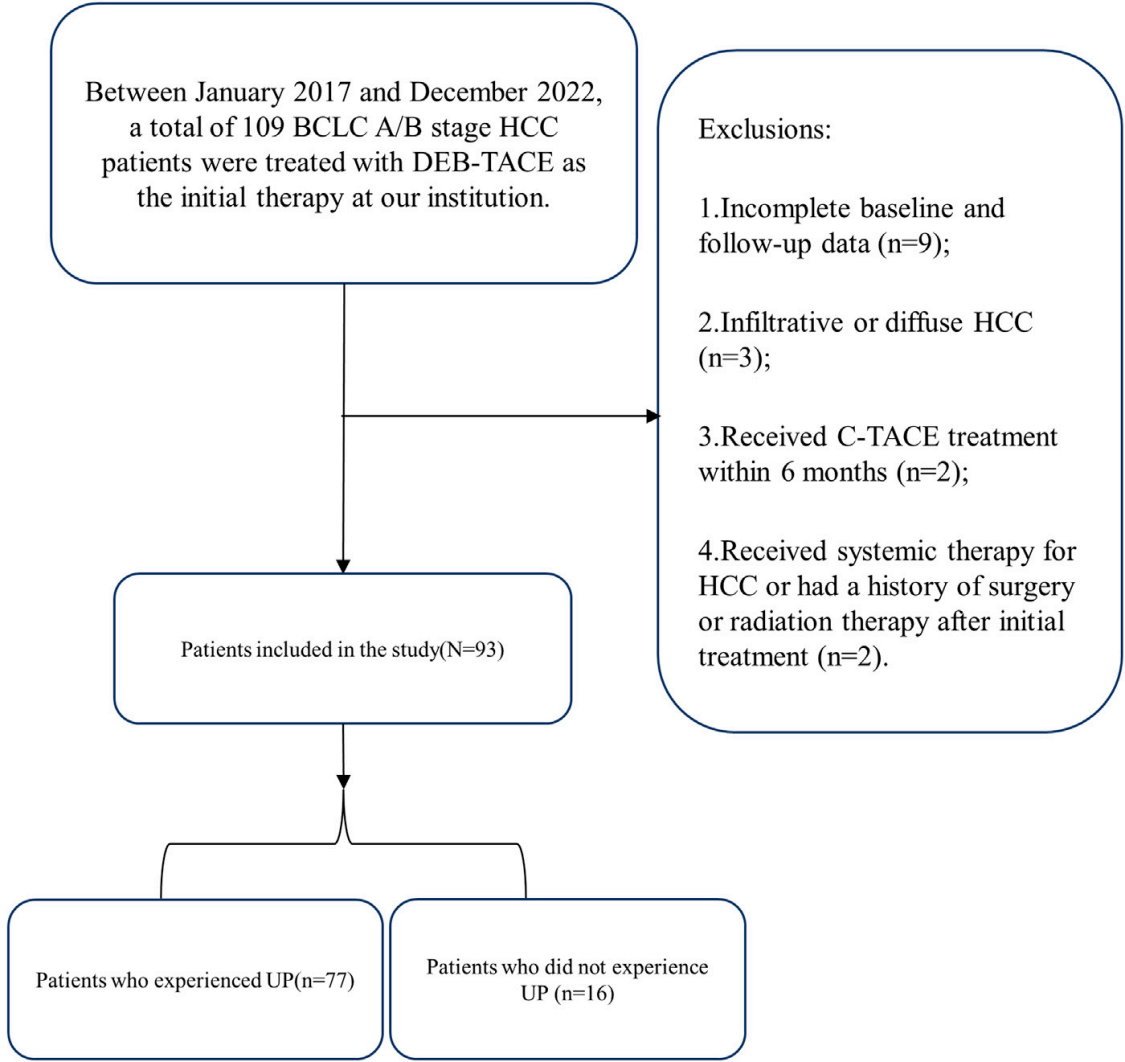
Flow chart shows exclusion criteria in patients with hepatocellular carcinoma.

**FIGURE 2:**
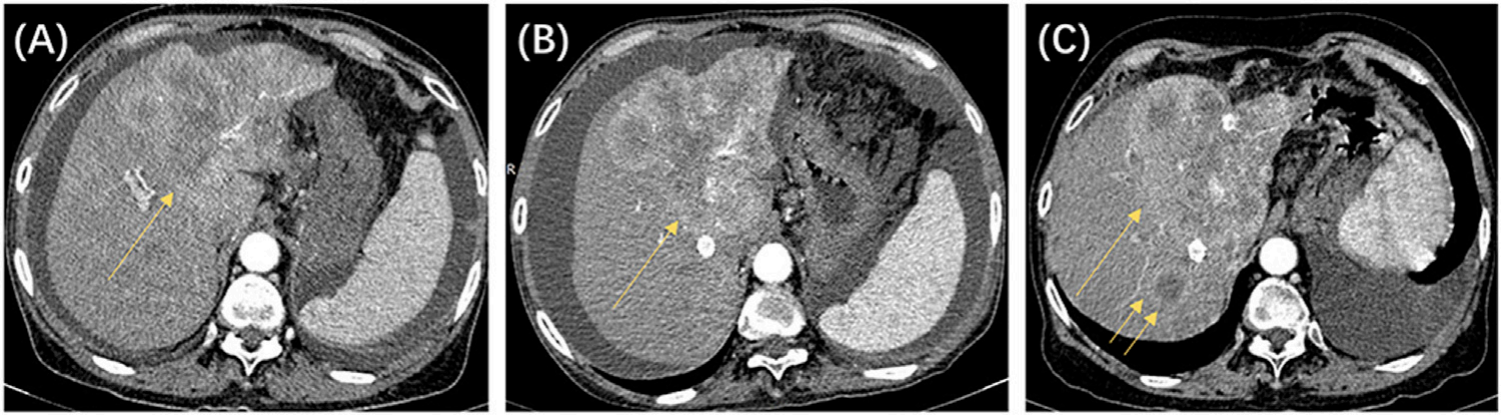
The patient was a 64-year-old woman, with a clinical diagnosis of hepatocellular carcinoma, Barcelona Clinic Liver Cancer stage B, and pre-treatment hepatic function of the Child–Pugh grade **(B)**. The above shows the contrast-enhanced CT images before and after locoregional interventional therapy. **(A)** Image taken before the initial drug-eluting beads transcatheter arterial chemoembolization (DEB-TACE) procedure, showing that the left liver lobe is full of blood-supplying, space-occupying lesions (single arrow). **(B)** CT image 8 weeks after the initial TACE procedure. Significant enhancement was still seen for the target tumor (single arrow). The target tumor was assessed to be stable disease based on modified response evaluation criteria in solid tumors (m-RECIST) criteria. **(C)** CT image 8 weeks after the second TACE procedure. Significant enhancement was still seen for the target tumor (single arrow), and new intrahepatic nodules were seen (double arrows). The target tumor was assessed to be progressive disease based on m-RECIST criteria. According to the untreatable progression (UP) criteria, the patient did not achieve an objective response after two DEB-TACE treatments and was determined to have UP.

**FIGURE 3:**
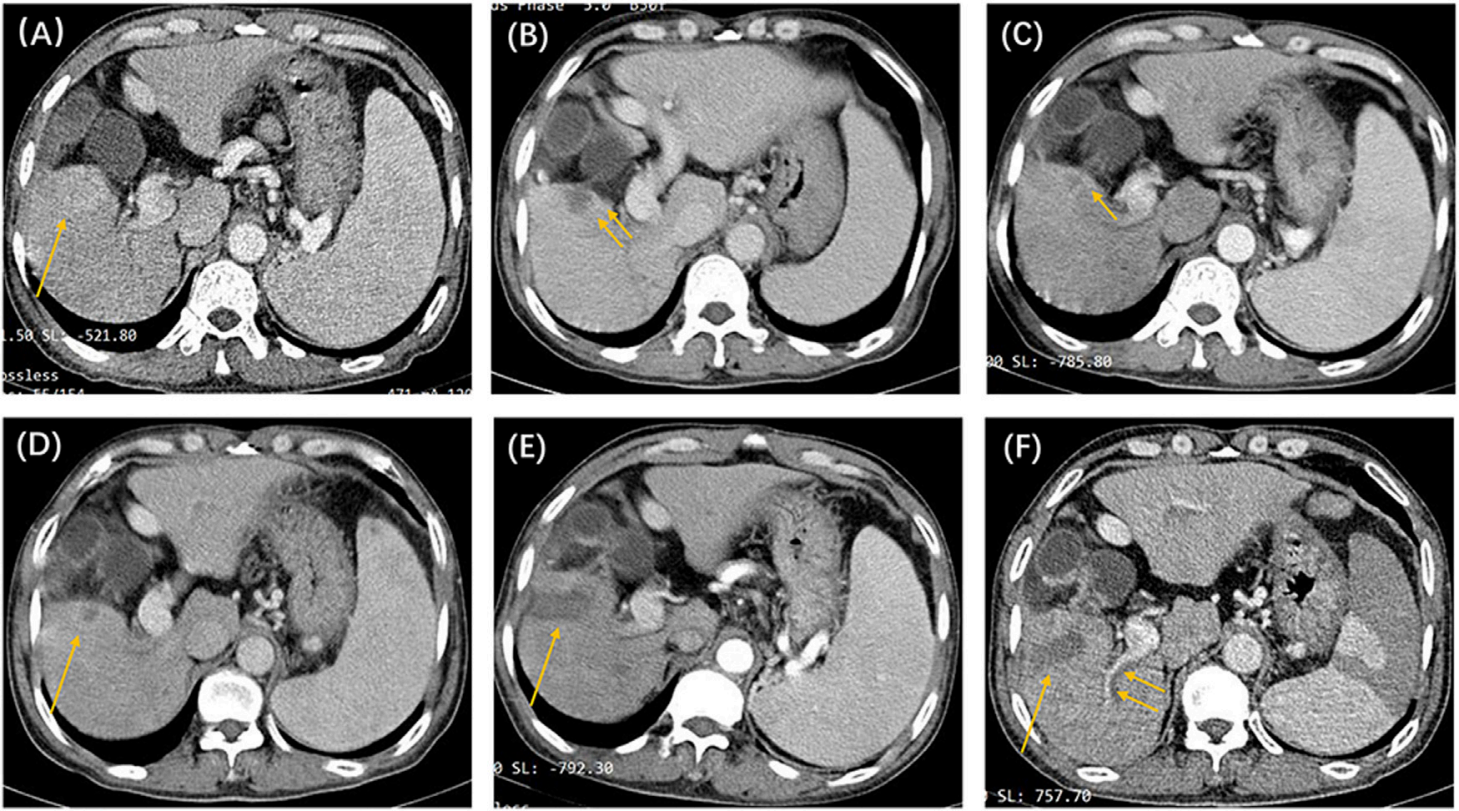
The patient was a 53-year-old man, with a clinical diagnosis of hepatocellular carcinoma, Barcelona Clinic Liver Cancer stage A, and pre-treatment hepatic function of the Child–Pugh grade **(A)**. The above shows the contrast-enhanced CT images before and after locoregional interventional therapy. **(A)** Image taken before the initial drug-eluting beads–transcatheter arterial chemoembolization treatment, showing that the right lower liver lobe is full of blood-supplying, space-occupying lesions (single arrow). **(B)** Image taken 6 weeks after the initial TACE procedure. Suspicious nodular enhancement lesions could be seen around the target tumor (double arrows). The target tumor was assessed to be partial response based on modified response evaluation criteria in solid tumors (m-RECIST) criteria. **(C)** CT image 8 weeks after the second TACE procedure. Suspected annular enhancement was still seen at the margin (single arrow). The target tumor was assessed to be stable disease based on m-RECIST criteria. **(D)** Periodic image review 16 weeks after the second TACE procedure. Marginal enhancement and suspected recurrence (single arrow) could be seen. Tumor microwave ablation was performed under CT guidance. **(E)** Image taken 6 weeks after the ablation procedure. The target tumor was assessed to be complete response (single arrow) based on m-RECIST criteria. **(F)** Periodic image review 14 weeks after ablation. The filling defect seen in the right hepatic portal vein branch (double arrow) was considered to be a vascular invasion. According to the untreatable progression (UP) criteria, the patient was ultimately deemed to have UP.

In this study, we identified 109 patients with HCC with BCLC stage A or B who initially underwent the DEB-TACE procedure at our hospital. After excluding 16 patients who did not meet the inclusion criteria, 93 patients were finally included in the study (Figure 1). Table 2 shows the baseline clinical and radiologic information of patients. The median follow-up period was 958 days (95% CI: 757–1,159). Based on the definition of UP, 77 (82.8%) patients developed UP; among them, 42 (54.5%) developed multiple intrahepatic nodules following treatment, and subsequent locoregional interventional therapy failed to induce tumor remission; 11 (14.3%) developed extrahepatic metastasis; 3 (3.9%) maintained SD after two continuous interventions (Figure 2); 13 (16.9%) developed vascular invasion (Figure 3); 2 (2.6%) had hepatic function progress from grade A or B to grade C; and 6 (7.8%) patients had primary tumor enlargement by >20%, and subsequent locoregional interventional therapy did not induce tumor remission.

**TABLE 2:** Baseline characteristics of the patients (N = 93).

Characteristics	Value	Monotherapy group	Combined group	*p*-value
Age (years)	58.33 ± 13.43	56.62 ± 13.42	60.33 ± 13.31	0.186
Serum albumin (g/L)	38.49 ± 5.88	38.19 ± 6.51	38.83 ± 5.12	0.361
Total bilirubin level (μmol/L)	18.37 ± 14.54	18.83 ± 18.07	17.84 ± 9.03	0.723
Platelet count (×10^9^ g/L)	188.70 ± 109.32	194.91 ± 128.04	181.49 ± 83.38	0.896
Maximum tumor diameter (cm)	6.35 ± 4.69	7.10 ± 5.12	5.48 ± 4.01	0.182
Tumor number	2.59 ± 2.74	2.86 ± 2.95	2.28 ± 2.48	0.301
Sex				0.251
Male	79 (84.95)	40(80.00)	39(90.70)	
Female	14 (15.05)	10(20.00)	4(9.30)	
Etiology				0.917
HBV	72 (77.42)	38 (76.00)	34 (79.07)	
Other	21 (22.58)	12 (24.00)	9 (20.93)	
BCLC stage				0.526
A	20 (21.51)	9 (18.00)	11 (25.58)	
B	73 (78.49)	41 (82.00)	32 (74.42)	
Child–Pugh grade				0.544
A	77 (82.80)	43 (86.00)	34 (79.07)	
B	16 (17.20)	7 (14.00)	9 (20.93)	
ALBI grade				0.844
1	39 (41.94)	20 (40.00)	19 (44.19)	
2 and 3	54 (58.06)	30 (60.00)	24 (55.81)	
AFP				0.272
≤400	72 (77.42)	36 (72.00)	36 (83.72)	
>400	21 (22.58)	14 (28.00)	7 (16.28)	
PT prolongation time score				0.456
1	83 (89.25)	46 (92.00)	37 (86.05)	
2	9 (9.68)	4 (8.00)	5 (11.63)	
3	1 (1.07)	0	1 (2.33)	
Tumor distribution				0.194
Monolobar	55 (59.14)	26 (52.00)	29 (67.44)	
Multilobar	38 (40.86)	24 (48.00)	14 (32.56)	
Drug-eluting bead particle size				0.521
70–150	9 (9.68)	4 (8.00)	5 (11.63)	
100–300	70 (75.27)	40 (80.00)	30 (69.77)	
300–500	14 (15.05)	6 (12.00)	8 (18.60)	
Vascular lake				1
Present	18 (19.35)	10 (20.00)	8 (18.60)	
Absent	75 (80.65)	40 (80.00)	35 (81.40)	
Capsule				1
Present	60 (64.52)	32 (64.00)	28 (65.12)	
Absent	33 (35.48)	18 (36.00)	15 (34.88)	
Type of enhancement				0.363
1 + 2	25 (26.88)	11 (22.00)	14 (32.56)	
3 + 4	68 (73.12)	39 (78.00)	29 (67.44)	
SACE				0.156
I	4 (4.30)	2 (4.00)	2 (4.65)	
II	12 (12.90)	10 (20.00)	2 (4.65)	
III	74 (79.57)	36 (72.00)	38 (88.37)	
IV	3 (3.23)	2 (4.00)	1 (2.33)	
Six-and-twelve score				0.126
≤6	33 (35.48)	17 (34.00)	16 (37.21)	
>6 and ≤12	36 (38.71)	16 (32.00)	20 (46.51)	
>12	24 (25.81)	17 (34.00)	7 (16.28)	
Initial response				0.442
Present	49 (52.69)	24 (48.00)	25 (58.14)	
Absent	44 (47.21)	26 (52.00)	18 (41.86)	
Best response				0.571
Present	79 (84.9)	41 (82.00)	38 (88.37)	
Absent	14 (15.1)	9 (18.00)	5 (11.63)	
Treatment regimen				
Monotherapy	50 (53.76)			
Combined	43 (46.24)			

There were 50 patients in the monotherapy group and 43 patients in the combined group. The baseline clinical and radiological features are presented in Table 2. In the monotherapy group, 47 (94%) patients developed UP, of which the median number of DEB-TACE treatments before UP was 2 (range: 2–6). In the combined group, 30 (69.8%) patients developed UP, of which the median frequency of DEB-TACE treatments before UP was 4 (range: 2–8). Furthermore, 49 (52.7%) patients achieved an initial response, of which 19 achieved CR and 30 achieved PR. Forty-four had no initial response, of which 38 maintained SD and 6 progressed to progressive disease (PD). Moreover, 36 (73.5%) patients with an initial response developed UP, and 41 (93.2%) patients without an initial response developed UP. Seventy-nine (85.0%) patients achieved the best response, of which 52 achieved CR and 27 achieved PR. Fourteen patients had no best response, of which 12 maintained SD and two progressed to PD. Sixty-three (79.7%) patients with the best response developed UP, and 14 (100%) patients without the best response developed UP (Table 3). Forty-one (82%) patients in the monotherapy group and 36 (83.7%) patients in the combined group achieved the best response. There were 4 (100%), 12 (100%), 59 (79.7%), and 2 (66.7%) patients with SACE levels I, II, III, and IV, respectively, who developed UP after the first TACE treatment.

**TABLE 3:** Initial and best response in detail.

		Best response				Total
		CR	PR	SD	PD	
Initial response	CR	19	0	0	0	19
	PR	15	15	0	0	30
	SD	14	12	12	0	38
	PD	4	0	0	2	6
Total		52	27	12	2	93

**FIGURE 4:**
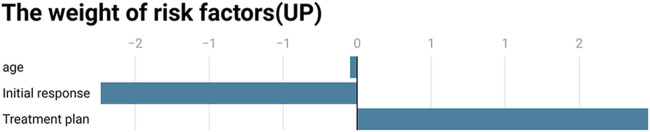
Visualization of the independent predictive factor weights for untreatable progression.

Univariate and multivariate logistic analyses revealed three independent risk factors associated with UP, which were age (odds ratio [OR]: 0.950, 95% CI: 0.904–0.999, *p* = 0.044); initial objective response (OR: 0.177, 95% CI: 0.041–0.759, *p* = 0.020); and treatment regimen (OR: 7.133, 95% CI: 1.705–29.842, *p* = 0.007). Among the three independent risk factors, treatment regimen had the highest contribution to the UP occurrence (Table 4; Figure 4).

**TABLE 4:** Logistic regression analysis of the predictive factors for untreatable progression.

	Univariate analysis		Multivariate analysis	
Characteristics	*p*-value	OR (95.0% CI)	*p*-value	OR (95.0% CI)
Age	0.032*	0.952 (0.911–0.996)	0.044*	0.950 (0.904–0.999)
Sex	0.230	2.233 (0.601–8.296)		
Etiology	0.688	1.322 (0.339–5.160)		
BCLC stage	0.709	1.271 (0.361–4.473)		
Child–Pugh grade	0.229	3.629 (0.444–29.675)		
ALBI grade	0.474	1.484 (0.504–4.372)		
Serum albumin	0.158	0.939 (0.860–1.025)		
Total bilirubin level	0.110	1.072 (0.985–1.166)		
Platelet count	0.291	1.003 (0.997–1.009)		
AFP	0.366	0.577 (0.175–1.900)		
PT prolongation time score	0.500	2.005 (0.266–15.100)		
Tumor distribution	0.059	3.611(0.952–13.696)		
Drug-eluting bead particle size	0.633	1.307 (0.436–3.919)		
Vascular lake	0.051	3.250 (0.994–10.627)		
Capsule	0.340	1.812 (0.534–6.151)		
Type of enhancement	0.297	1.832 (0.588–5.710)		
SACE	0.058	0.201(0.038–1.054)		
Maximum tumor diameter	0.198	1.098(0.953–1.265)		
Tumor number	0.070	1.829 (0.952–3.513)		
Six-and-twelve score	0.061	0.476 (0.219–1.034)		
Initial response	0.019*	0.203 (0.053–0.768)	0.020*	0.177 (0.041–0.759)
Best response	0.999	0.000 (0.000–)		
Treatment regimen	0.005*	6.789 (1.784–25.832)	0.007*	7.133 (1.705–29.842)

Bold is statistically significant factors.

**FIGURE 5:**
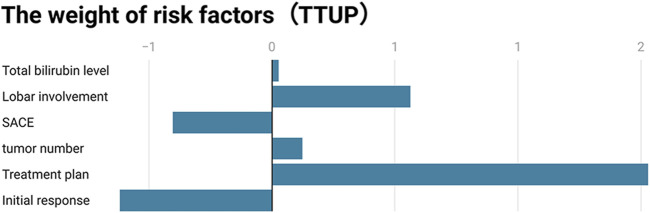
Visualization of the independent factor weights for time to untreatable progression.

Univariate Cox regression analysis found that total bilirubin, SACE classification, tumor distribution, maximum tumor diameter, number of tumors, six-and-twelve tumor burden group, initial response, and treatment regimen were variables that significantly influenced TTUP. Multivariate Cox regression confirmed that total bilirubin (HR: 1.029, 95% CI: 1.011–1.047, *p* = 0.002); tumor distribution (HR: 1.752, 95% CI: 1.044–2.938, *p* = 0.034); SACE classification (HR: 0.668, 95% CI: 0.452–0.987, *p* = 0.043); number of tumors (HR: 1.130, 95% CI: 1.039–1.229, *p* = 0.004); initial objective response (HR: 0.539, 95% CI: 0.322–0.903, *p* = 0.019); and treatment regimen (HR: 4.615, 95% CI: 2.621–8.126, *p* < 0.001) were independent variables affecting TTUP. Among the six TTUP-related independent variables, treatment regimen had the highest impact (Table 5; Figure 5).

**TABLE 5:** Cox regression analysis of the predictive factors for time to untreatable progression.

	Univariate analysis		Multivariate analysis	
Characteristics	*p*-value	HR (95.0% CI)	*p*-value	HR (95.0% CI)
Age	0.115	0.987 (0.971–1.003)		
Sex	0.802	1.090 (0.558–2.127)		
Etiology	0.353	1.291 (0.753–2.211)		
BCLC stage	0.070	1.681 (0.959–2.949)		
Child–Pugh score	0.852	0.947(0.537–1.672)		
ALBI grade	0.191	1.373 (0.854–2.208)		
Serum albumin	0.588	0.988 (0.946–1.032)		
Total bilirubin level	<0.001*	1.032 (1.014–1.050)	0.002*	1.029 (1.011–1.047)
Platelet count	0.135	1.002 (0.999–1.005)		
AFP	0.994	1.002 (0.576–1.743)		
PT prolongation time score	0.699	1.127 (0.615–2.067)		
Tumor distribution	0.021*	1.700 (1.082–2.670)	0.034*	1.752 (1.044–2.938)
Drug-eluting bead particle size	0.837	1.044 (0.693–1.573)		
Vascular lake	0.223	1.467 (0.791–2.720)		
Capsule	0.153	1.403 (0.881–2.235)		
Type of enhancement	0.083	1.604 (0.941–2.735)		
SACE	0.017*	0.654 (0.461–0.928)	0.043*	0.668 (0.452–0.987)
Maximum tumor diameter	0.017*	1.060 (1.060–1.113)	0.735	
Tumor number	0.001*	1.129 (1.054–1.211)	0.004*	1.130 (1.039–1.229)
Six-and-twelve score	0.002*	0.632 (0.471–0.848)	0.767	
Initial response	0.003*	0.496 (0.315–0.782)	0.019*	0.539 (0.322–0.903)
Best response	0.001*	0.342 (0.184–0.636)	0.430	
Treatment regimen	<0.001*	4.013 (2.359–6.827)	<0.001*	4.615 (2.621–8.126)

Bold is statistically significant factors.

**FIGURE 6:**
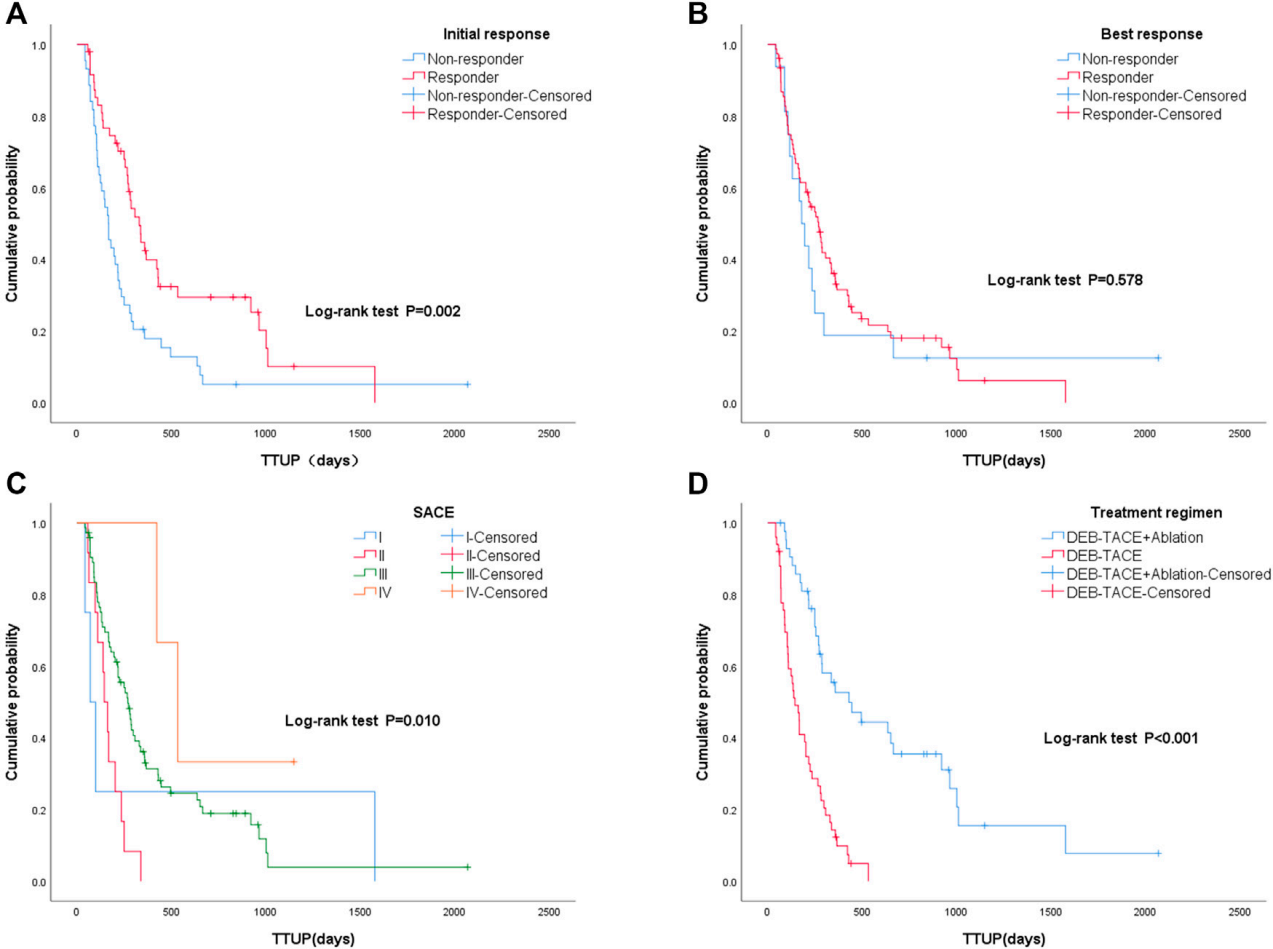
Kaplan–Meier curves of different groups: **(A)** with initial response and without initial response; **(B)** with best response and without best response; **(C)** different Subjective Angiographic Chemoembolization Endpoint classification levels (I, II, III, IV) during the first TACE procedure; **(D)** different treatment regimens: monotherapy group (drug-eluting beads–transcatheter arterial chemoembolization [DEB-TACE] treatment alone) and combined group (DEB-TACE and sequential ablation).

The median TTUP of patients with UP was 249 days (95% CI: 188–310). Among them, the median TTUP of the monotherapy group was 144 days (95% CI: 95–193), and the median TTUP of the combined group was 429 days (95% CI: 204–654) (*p* < 0.05). The TTUP of patients with an initial response was 329 days (95% CI: 259–399), which was significantly longer than those without an initial response (166 days; 95% CI: 128–204) (*p* < 0.05). Among them, the median TTUP of CR, PR, SD, and PD were 366, 282, 167, and 103 days, respectively (*p* < 0.05). However, the TTUP of patients with the best response was 279 days (95% CI: 240–318), which was significantly longer than those without the best response (130 days; 95% CI: 36–224) (*p* < 0.05). Among them, the median TTUP of CR, PR, SD, and PD were 338, 172, 167, and 70 days, respectively (*p* < 0.05). Among different SACE levels, the median TTUP for grades I, II, III, and IV was 70, 144, 269, and 533 days, respectively (*p* < 0.05) (Figure 6).

Kappa agreement test results showed that the radiologic diagnosis of tumor distribution (K = 0.956), vascular lake (K = 0.868), capsule (K = 0.885), enhancement type (K = 0.945), SACE (K = 0.860), initial response (K = 0.850), and best response (K = 0.817) were consistent.

## 4 Discussion

The TACE procedure can moderately extend the survival of intermediate- and advanced-stage HCC patients. However, multiple TACE treatments may result in treatment failure or disease progression and liver impairment, resulting in a poor prognosis. Therefore, the balance between the benefits and side effects of repeated locoregional interventional therapy must be given due consideration. Currently, UP is most widely considered the best marker for indicating treatment failure or disease progression in HCC patients and is thus commonly used to decide whether locoregional interventional therapy should be discontinued in favor of advanced treatment regimens (Bruix et al., 2011; Raoul et al., 2011).

Although OS is the gold standard for measuring tumor response to treatment, numerous treatment options can mitigate HCC progression; hence, it is impractical to use OS as the study endpoint for locoregional interventional therapy. Therefore, there is a need for a surrogate endpoint for OS (Kudo et al., 2020b). “Disease progression” in the traditional sense may be controlled through a subsequent successful TACE procedure. Analysis of prior findings (Izumoto et al., 2017) found that the time from the first TACE procedure to TACE refractoriness, that is, time to TACE progression, has a poor actual impact on OS (R = 0.527, *p* < 0.001) and is not a good surrogate endpoint. TTUP mainly describes the time from the start of treatment to the onset of UP and can indicate when to switch treatment regimens for the patient. Labeur et al. (2019) previously reported that TTUP was strongly correlated with OS (R = 0.816, *p* < 0.001) and can be used as a surrogate endpoint for OS. Recently, TTUP was used to assess the prognosis of patients with intermediate-to-advanced HCC treated with TACE in many studies (Hsu et al., 2012; Kudo et al., 2022). Hence, this study analyzed variables that influence UP and TTUP to predict the onset of UP and provided recommendations for switching to more advanced treatment regimens.

In a previous retrospective analysis, Zhang et al. (2023) found that only 42 (20.4%) out of 206 HCC patients developed UP, and the median follow-up was 97 days (95% CI: 58–217 days). The study by Labeur et al. (2019) found that 116 out of 166 HCC patients developed UP, and the median follow-up period was 40.5 months (95% CI: 27.6–53.3 months). In this study, 77 (82.8%) patients developed UP, and the median follow-up period was 958 days (95% CI: 757–1159 days). The proportion of patients who developed UP was significantly higher than in the study by Zhang et al. and aligned better with the study by Labeur et al. This may be related to the follow-up duration. HCC is highly heterogeneous, with a tendency for multifocal development, recurrence, and metastasis. Although most patients can achieve tumor remission after TACE treatment, disease progression will ultimately occur. Therefore, the probability of developing UP increases with the follow-up duration.

This study included patients who underwent TACE and sequential ablation treatments for analysis based on the recommendations proposed in the BCLC guidelines and actual clinical practice procedures. Previous meta-analyses showed that the recurrence-free survival and OS of patients who received TACE and sequential ablation treatments were higher than those of patients who underwent TACE treatment alone (Liu et al., 2020; Jiang et al., 2021). The study by Liu et al. (2020) found that the median time to progression for patients who received TACE alone was 4.00 (3.00–5.00) months, while the median time to progression of patients who received TACE and a sequential ablation treatment regimen was 9.13 months (6.64–11.62 months; *p* < 0.001). In this study, the authors suggested that TACE and sequential ablation was a safe and valid treatment regimen for unresectable HCC. Our results also validated this conclusion: the incidence of UP in the monotherapy group was significantly higher than that in the combined group (94% vs. 69.8%), and the risk of UP in the monotherapy group was also increased by 7.33 times (95% CI: 1.705–29.842) compared to that in the combined group. In addition, the median TTUP of the monotherapy group was significantly shorter than that of the combined group (144 days vs. 429 days, *p* < 0.05). These findings may originate from incomplete tumor necrosis from the TACE procedure alone and the simultaneous TACE-induced increases in vascular endothelial growth factor, thereby promoting tumor progression (Kudo et al., 2020a).

Multiple TACE treatments are known to cause a decline in hepatic function. A study recommended TACE and a sequential ablation treatment regimen for unresectable HCC, as the elimination of tumors following ablation can be enhanced by TACE (Hirooka et al., 2018). The mechanism by which this occurs is likely that it is difficult for ablation alone to completely eliminate tumors >3 cm, and incomplete ablation may result in a risk of tumor recurrence. In China, patients with unresectable HCC often have tumor diameters >3 cm. Angiography can be performed first to understand the extent of lesion distribution and the formation of surrounding sub-lesions. TACE treatment can shrink the tumor and preliminarily decrease tumor burden. Subsequent combination with ablation to treat residual tumor can increase the recurrence-free survival rate and improve patient prognosis.

This study found that the tumor initial response is an important risk factor affecting UP occurrence and is also an independent variable influencing TTUP, which may be because of the timeliness of the initial response (Xia et al., 2022b; Zhang X et al., 2024). This study found that the ratio of patients with an initial response who developed UP was lower than that of patients without an initial response (73.5% vs. 93.2%). The median TTUP of patients with an initial response was 329 days, while the median TTUP of patients without an initial response was 166 days (*p* < 0.05). This means that the TTUP of patients with an initial response after the first DEB-TACE was significantly increased compared with that of those without an initial response. An earlier study (Lee et al., 2017) found that the early treatment response of intermediate-stage HCC patients after TACE treatment was significantly correlated with OS and that the median OS of patients with early treatment response after the TACE procedure was longer than for those without early treatment response (45.9 months vs. 14.4 months, *p* < 0.05). Maesaka et al. (2020) showed that the initial response following the TACE procedure is an important factor affecting hepatic function changes in HCC patients. When there was no response to initial TACE treatment, patients were recommended molecular targeted (MTA) therapy early to avoid hepatic function decline. Another retrospective study on 84 HCC patients found that the TTUP of patients with an initial response was 9.23 months (95% CI: 8.17–11.13), while patients without an initial response had a TTUP of 2.23 months (95% CI: 1.63–2.93) (Wang et al., 2020), which is shorter than the present study. This may be because their study analyzed patients who underwent TACE treatment, while our study included patients who underwent DEB-TACE and sequential ablation treatments into the analysis, which can prolong TTUP.

The study by Kim et al. (2015) found that initial response and best response are strongly correlated with OS. Choi et al. (2014) found that the best response of intermediate-stage HCC patients during continuous TACE treatment was predictive of patient survival. In this study, patients with no best response after locoregional interventional therapy had a higher probability of UP than those with the best response (100% vs. 79.7%). Simultaneously, there was no statistical significance between the best response and UP occurrence (*p* > 0.05). The median TTUP of patients with the best response was longer than in those without the best response (279 vs. 130 days, *p* < 0.05); the correlation with TTUP was poor. This study was unable to fully demonstrate the effects of best response on patient prognosis. This is mainly because TTUP was used as a surrogate endpoint for OS to evaluate the efficacy of locoregional interventional therapy in patients; another potential reason could be the low sample size and uneven sample distribution in the study.

Our results showed that the SACE classification significantly affected the TTUP of patients. The probability of developing UP when the degree of embolization was SACE levels I, II, III, and IV was 100%, 100%, 79.7%, and 66.7%, respectively. The TTUP of SACE levels III + IV was longer than for levels I + II (269 days and 533 days vs. 70 days and 144 days). Jin et al. (2011) found that patients who underwent intermediate embolization (SACE level III) had better survival advantages than those who underwent excessive embolization (SACE level IV). This conclusion differs from the results of our study. The main reason is that most patients in our study underwent SACE level III treatment (n = 74), and only three patients underwent SACE level IV treatment. Therefore, our results cannot demonstrate the differences between intermediate embolization and excessive embolization. Furthermore, the study of Habbel et al. (2019) indirectly proved the effects of SACE classification on patient prognosis. Their study found that although the SACE classification did not directly affect OS, it affected the local and overall tumor OR, which indirectly affected the OS and progression-free survival of patients.

This study found that tumor distribution classification significantly affected the TTUP of patients. Multilobar tumors have complex nourishing blood vessels, particularly for HCC in the caudate lobe and left liver lobe as central anastomosis of the hepatic blood vessel variants and collateral vessels, which makes the TACE procedure further challenging (Vesselle et al., 2016), thereby making it difficult to completely eradicate the tumor. In addition, nontumor liver tissues are more susceptible to the effects of embolization than tumor tissues (Bannangkoon et al., 2018), thereby decreasing the hepatic function and shortening the TTUP of patients. Moreover, it was found that total bilirubin and the number of tumors were significantly correlated with TTUP, which was consistent with the variables affecting patient survival in the mHAP-III scoring model constructed by Cappelli et al. (2016). Total bilirubin reflects hepatic function reserve, and hepatic function is decreased when total bilirubin increases, resulting in a poorer prognosis (Lee et al., 2014). The studies by Hu et al. (2011) and Wang et al. (2020) found that the number of tumors was significantly correlated with survival, and the mechanism may be that tumor burden affects the efficacy of DEB-TACE therapy, which in turn affects the prognosis of patients. In addition, this study also found that older patients have a lower risk of developing UP than younger patients (OR: 0.950, *p* = 0.044). This may be because the younger patients tended to show advanced tumor characteristics (large maximum tumor diameter, increased number of tumors, microvascular invasion, and poorly differentiated tumor cells) compared to older patients (Diao et al., 2021).

This study has some limitations. First, it was a single-center retrospective study with a potential risk of selection bias. In addition, the data volume is low, and the data distribution is uneven. Hence, the data may not be generally representative, and multicenter data will be included in subsequent studies. Second, this study only analyzed TACE and ablation treatments, and other additional treatments were not included. There was no stratification of different ablation treatment regimens, which could have affected the results. Third, our study only analyzed TTUP and did not collect the patient OS data. Hence, we were unable to comprehensively understand patient’s prognosis. Fourth, we only collected the SACE classification of the first DEB-TACE procedure and did not analyze the SACE classification of subsequent TACE procedures. Last, there was a strong observer dependence on subjective variables.

## 5 Conclusion

The absence of an initial response, use of DEB-TACE treatment alone, or younger age was considered risk factors that increased the risk of developing UP in HCC patients. Initial response, SACE classification, treatment regimen, total bilirubin, number of tumors, and tumor distribution were all important variables affecting the patient’s TTUP. Following locoregional interventional therapy, the initial response affected UP occurrence and the patient’s TTUP more than the best response. We believe that the previous results can provide recommendations for deciding the timing for treatment regimen switching for patients and hope that the two concepts of UP and TTUP can be further used in decision-making for treatment regimens in HCC patients for future studies.

## Data Availability

The original contributions presented in the study are included in the article/Supplementary Material; further inquiries can be directed to the corresponding author.

## Abbreviations

ALBI, albumin–bilirubin; AFP, alpha fetoprotein; BCLC, Barcelona Clinic Liver Cancer; CI, confidence interval; CR, complete response; C-TACE, conventional-TACE; CSCO, Chinese Society of Clinical Oncology; DEB-TACE, drug-eluting beads–transcatheter arterial chemoembolization; ECOG, Eastern Cooperative Oncology Group; HCC, hepatocellular carcinoma; HR, hazard ratio; JSH, Japan Society of Hepatology; m-RECIST, modified response evaluation criteria in solid tumors; MTA, molecular targeted; OS, overall survival; OR, odds ratio; PD, progressive disease; PR, partial response; PT, prothrombin time; SACE, Subjective Angiographic Chemoembolization Endpoint; SD, stable disease; TACE, transcatheter arterial chemoembolization; TTUP, time to untreatable progression; UP, untreatable progression.
